# Fast occlusion-based point cloud exploration

**DOI:** 10.1007/s00371-021-02243-x

**Published:** 2021-07-28

**Authors:** Mohamed Radwan, Stefan Ohrhallinger, Michael Wimmer

**Affiliations:** TU Wien, Vienna, Austria

**Keywords:** 3D navigation, Real-time processing, Occlusion

## Abstract

Large-scale unstructured point cloud scenes can be quickly visualized without prior reconstruction by utilizing levels-of-detail structures to load an appropriate subset from out-of-core storage for rendering the current view. However, as soon as we need structures within the point cloud, e.g., for interactions between objects, the construction of state-of-the-art data structures requires *O*(*NlogN*) time for *N* points, which is not feasible in real time for millions of points that are possibly updated in each frame. Therefore, we propose to use a surface representation structure which trades off the (here negligible) disadvantage of single-frame use for *both output-dominated and near-linear construction time in practice*, exploiting the inherent 2D property of sampled surfaces in 3D. This structure tightly encompasses the assumed surface of unstructured points in a set of bounding depth intervals for each cell of a discrete 2D grid. The sorted depth samples in the structure permit fast surface queries, and on top of that an occlusion graph for the scene comes almost for free. This graph enables novel real-time user operations such as revealing partially occluded objects, or scrolling through layers of occluding objects, e.g., walls in a building. As an example application we showcase a 3D scene exploration framework that enables fast, more sophisticated interactions with point clouds rendered in real time.

**Supplementary Information:**

The online version supplementary material available at 10.1007/s00371-021-02243-x.

## Introduction

A variety of current sensors allows acquiring large scenes as dense point clouds, and state-of-the-art methods can render such huge 3D data in real time. Billion-sized point clouds can be inspected using hierarchical structures that select frame-varying levels of detail (LOD) as a few million points subset within the viewing frustum for rendering. As interactive 3D applications become more common, visualization is not enough, and the demand increases to interact with and explore such point clouds, which can quickly change between frames. In this paper, we investigate interactive handling and querying changing subsets of point clouds beyond simple visualization and navigation.

In order to make the per-frame construction and querying of a data structure fast enough, we exploit both the fact that for changing subsets of a point cloud, a view-dependent structure is sufficient, and that the inherent 2D property of samples of a surface results in low depth complexity for its projection. A related method is *thickened layered depth images* (TLDI) [29], which is constructed subsequently for each depth layer, and extends the depth value of LDIs [19] to a bounding interval (for a more detailed description, see Sect. 3.1). We propose to extend it to a more generic and efficient *discrete depth structure* (DDS) that re-samples the point cloud at a 2D grid and generates a list of intervals per cell, orthogonal to the grid, for all depth layers *in the same pass*, thus drastically reducing the runtime of several passes on the same input data that TLDI requires. In case this grid is aligned with the view plane, these lists correspond to depth intervals encapsulating the surface along the box projected by the cell into 3D, and thus the surface can be approximated by the centers of these intervals. The construction time of the DDS is not a function of points but rather of fragment count (see Sect. 3.2). For input point clouds with area-covering splat radii, the number of fragments is always proportional to the grid size, making its runtime mostly output-sensitive. We also explain a modified pipeline to label the depth intervals by object ID while constructing the DDS, for applications where depth bounds of different objects should be distinguished. This requires just an additional sorting pass, whereas the TLDI has to be *constructed for each object separately* in such cases.

A common challenge in exploring immersive scenes is to find occluded objects. As occlusions are view-dependent, occlusion relations between the scene objects can easily be extracted from the sorted view-aligned depth intervals of the DDS. This is similar to casting rays perpendicular to (and at the centers of the cells of) the 2D grid on which the DDS is built, where the discretization guarantees that all parts of the surface relevant to the current view is considered. Once an occlusion graph has been built for a view, occluders of an object or a set of objects can be determined much faster because of the much coarser (object) granularity of the graph compared to considering individual points. We provide an exploration tool, based on occludee revealing, to aid users with quickly understanding a scene and the spatial relations between its objects. This tool is coupled with a rendering structure, specifically Potree [34], to form a complete framework for both rendering and exploring huge point clouds. Potree is responsible for selecting a subset of points for rendering the current view, while the processing module builds the DDS on the selected points and constructs the occlusion graph used by the exploration component.

**Fig. 1:**
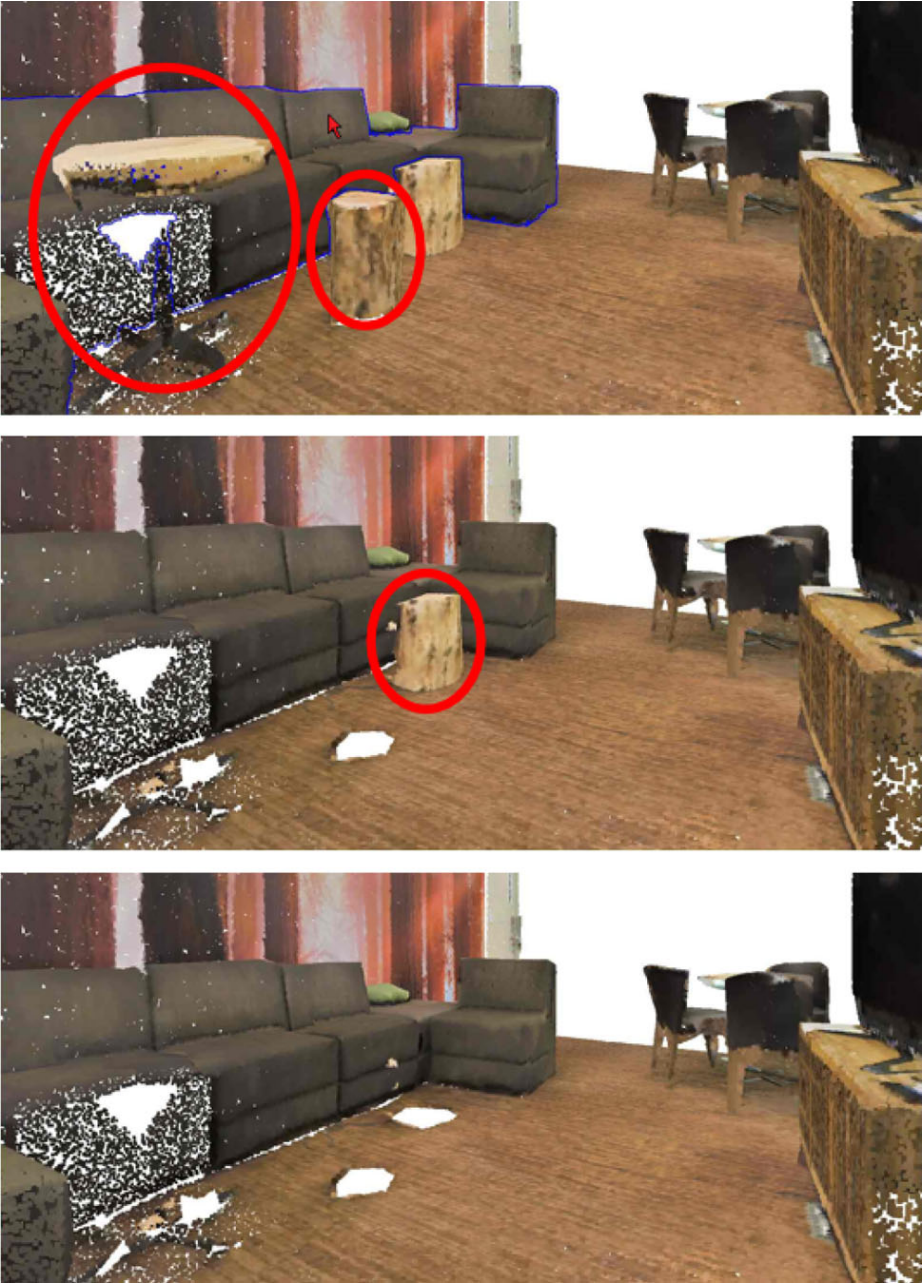
Interactively browsing occluded objects: Occluders of a selected object are arranged in visibility layers for browsing through them, using the *Browse Occluders* function. Top: The couch is selected (border shaded blue). Center and Bottom: First and second visibility layers of occluders (encircled red) of the couch, respectively, are removed in order to expose the entire couch

Our contributions in this paper are:

- The DDS: A tight surface-bounding structure for point clouds with constant time queries which generalizes the TLDI [29] and constructs much faster, in the same pass for all depth layers
- As an application, novel intuitive and real-time operations for effective point cloud explorations which are enabled by using the DDS (see Fig. 1)

We demonstrate these contributions in a framework that can both visualize and explore out-of-core point clouds in real time. Furthermore, the DDS can also be used for accelerating queries such as k-nearest neighbors, ray casting, collision and change detection.

In Sect. 2 we describe related work, then the technical details of our proposed DDS in Sect. 3, before presenting the framework in Sect. 4. We evaluate the performance in Sect. 5 and conclude in Sect. 6, together with an outlook towards future work.

## Related work

*Multi-layers Structures:* Algorithms based on projecting 3D models into multiple layers in screen space have been used in several applications such as ray tracing, collision detection, and transparency. Everitt [15] introduced *depth peeling*, where geometric data are repeatedly projected to extract one depth layer at a time. *T*he illustration buffer introduced by Carnecky et al. [5] is a view-aligned structure that stores all depth layers through a single data projection, and is used to control transparency at objects silhouettes for enhanced illustrations. Hofmann et al. [21] perform screen-space ray marching on a hierarchical multi-layer structure, that merges fragments to form lower resolutions.

*L*ayered Depth Images (LDIs) [35], which are basically depth layers, were used to approximate volumes and detect collisions [18], and to model collisions responses [16]. All these methods and structures process and sample continuous surfaces in the form of polygonal meshes. Our method allows for including more data representations, such as sampled point sets. We define the underlying surface, and resample it uniformly.

Radwan et al. [29] based their *T*hickened Layered Depth Images (TLDI) on the LDIs to detect collisions between point clouds by enveloping the points with an assumed thickness derived from point density. Their approach to extract depth images is similar to depth peeling, augmented by stages to merge depth intervals. Our proposed DDS is based on similar definitions and provides the same purposes, but builds the depth bounds much more efficiently and independent on layers complexity so that occlusions graphs can be determined by a small fraction of the time, making it very near-linear in practice.

*Occlusion Management:* Many occlusion management techniques have been proposed to discover fully and partially hidden objects in visualizations. Elmqvist and Tsigas [13] recognized five broad design patterns in such techniques, namely volumetric probes, multiple views, virtual X-ray, tour planners and projection distorters. In the *m*ultiple views pattern, different views and perspectives of the virtual world are presented, such as the hand-held world copy WorldInMiniature [36], worldlets [14], visibility widgets [30], and multi-perspective images [40]. The *v*olumetric probes find a hidden object among occluders using a probe object, such as Depth Ray or 3D Bubble Cursor [38], possibly transforming or distorting the occluders [3, 7], or rearranging cluttered objects in a planar view to select the desired one such as SQUAD [24] and EXPAND [6]. *V*irtual x-rays turn objects transparent or semi-transparent in order to reveal occluded items [11, 25]. All these techniques assume that the objects have a priori well-defined surfaces, and that they are arranged in a tree-like structure, in order to find occlusions and order along depth efficiently via ray casting. In our approach, this information is extracted from the DDS, which is built in real time and approximates the surfaces of unstructured point clouds. We reveal partially occluded objects by hiding the occluders; however, different visualization (e.g., transparency), can be easily integrated in our system.

Mossel and Koessler [25] also target dense point clouds. However, their approach allows users to locate just a single object, walk closer to it along a cut plane, and segment it with a region growing algorithm. Our approach structures the whole scene in real time, and allows for a faster revealing of many objects, without changing the viewpoint.

A different approach to handle occlusions was presented by Eisemann et al. [12], where they cut meshes into layers based on visibility. This method is used to convert 3D geometry into 2D vector graphics, and is most useful to cut self-occluded objects.

*Bounding structures:* Convex bounding volumes (e.g., convex hulls, spheres, bounding boxes, *k*-DOPs) are used as efficient shape representation in many applications to speed up queries and intersection tests. Other coarse bounding meshes—sometimes called “cages”—fit highly non-convex shapes tighter and perform faster than convex volumes, such as nested cages [32], bounding proxies [4], and others [8, 10, 33, 41]. Those structures bound the shape volume, while the DDS is a tight bound of the surface, making it suitable for representing the different surface layers of the shape from a specific view, as well as handling shapes which are incomplete or have boundaries.

*Large point sets sampling:* Large-scale point clouds are usually rendered using hierarchical levels-of-detail structures, which sub-samples them into smaller representative subsets for actual rendering. Rusinkiewicz and Levoy [31] introduced the first hierarchical structure for rendering points, and Dachsbacher et al. [9] proposed a sequential version of it for efficient processing by GPUs. Several following approaches proposed multi-resolution tree structures [2, 17, 26, 34, 37, 39]. Our framework integrates the state-of-the-art multi-resolution Potree [34] with the DDS for real-time scene rendering and exploring.

## Discrete depth structure

The DDS is based on similar definitions as the TLDI [29], and stores the same information, but it is more generically applicable, and we present a much more efficient method to construct it by combining its multiple passes on input data into a single one. Section 3.1 reviews the common theoretical basis, and Sect. 3.2 explains the new, more efficient construction pipeline.

### Discrete surface bound

**Fig. 2:**
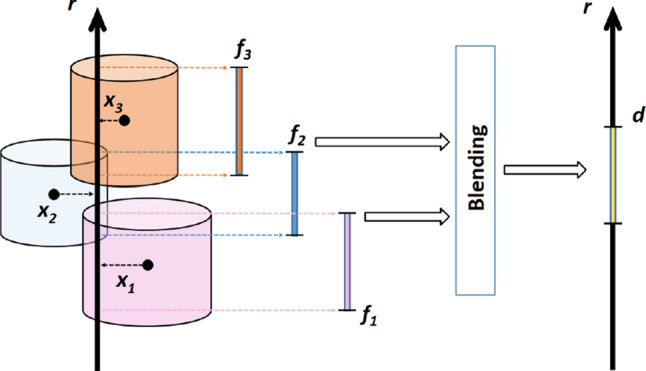
The ray *r* intersects three overlapping cylinders, generating three fragment intervals, $f_{1,2,3}$, which are blended into the depth bound interval (DBI) *d*

We now repeat some definitions [29], partly adapted: They define a tight cover of spheres $\Omega$ to enclose a set of points and their underlying surface $\Sigma$. The intersection of spheres defines the connectivity between points, which is simplified to the intersection of disks due to their projection onto a plane, and overlaps in the direction orthogonal to that plane. They refer to that projection direction as depth, and we also use it that way in our proposed method. In 3D, this union of disks corresponds to a union of cylinders (termed $\Omega '$) whose bases are aligned to the projection plane. A ray perpendicular to that projection plane intersects each cylinder in a 1D segment along the depth which is called “fragment interval.” For an equal thickness of the boundary in view direction, all overlapping depth intervals are blended. Blending computes both start and end of the result interval as a weighted mean of the overlapping intervals’ start and end values. The weight should be inversely proportional to the distance *x* between the ray and the point enclosed by the cylinder, projected onto the view plane. Therefore, we choose as weight the Gaussian function $w_i=e^{-x_i^2}/ \sum _i e^{-x_j^2}$ for fast decay. We call these resulting depth intervals (blended from overlapping cylinders, or stemming from single cylinders) “depth bound intervals,” DBI, and $\hat{\Omega }$ is defined as the union of those DBIs. Figure 2 illustrates the concept of cylinder intersections, blending and DBIs.

**Fig. 3:**
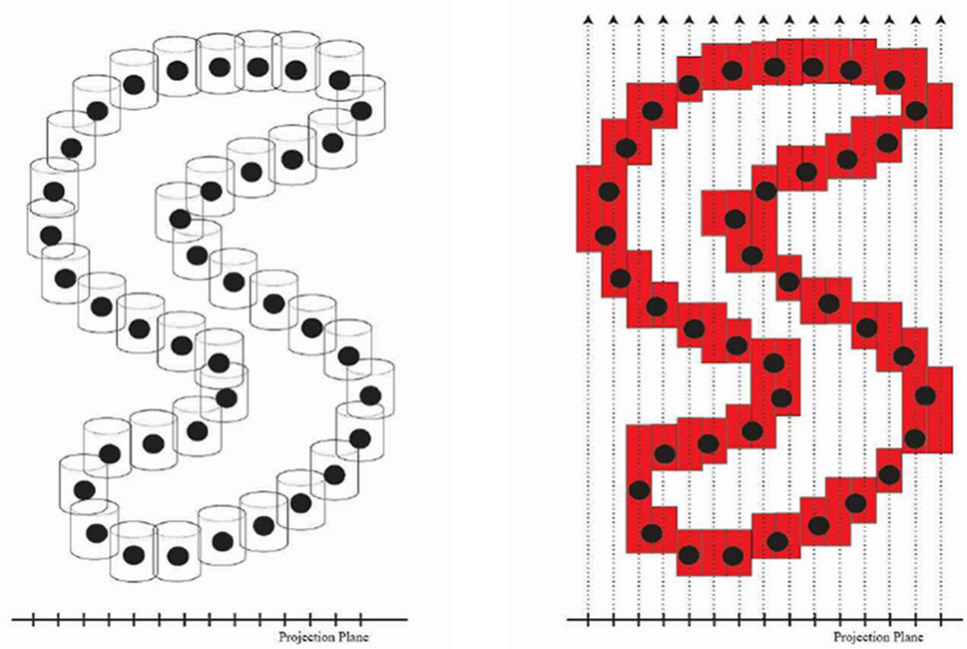
Left: The surface sample points are enclosed in the cylinder cover $\Omega '$. Right: Cylinders’ depth ranges along view-aligned rays are blended into depth bound intervals $\hat{\Omega }$

Then, they approximate $\Sigma$ with the smooth surface $\hat{\Sigma }$, which they define as bounded by the union of cylinders and passing through the centers of the DBIs of $\hat{\Omega }$. We call these centers “depth samples.” The DBIs can also be considered as confidence intervals for the depth samples’ positions.

In order to enable hole-free reconstruction of the surface, the cylinders have to overlap. Thus, the radii of the cylinders have to be estimated from the sampling density, either per point using k-nearest neighbors search, or as a global parameter from the minimum sample distance, to avoid the search and preserve the time complexity advantage.

**Fig. 4:**
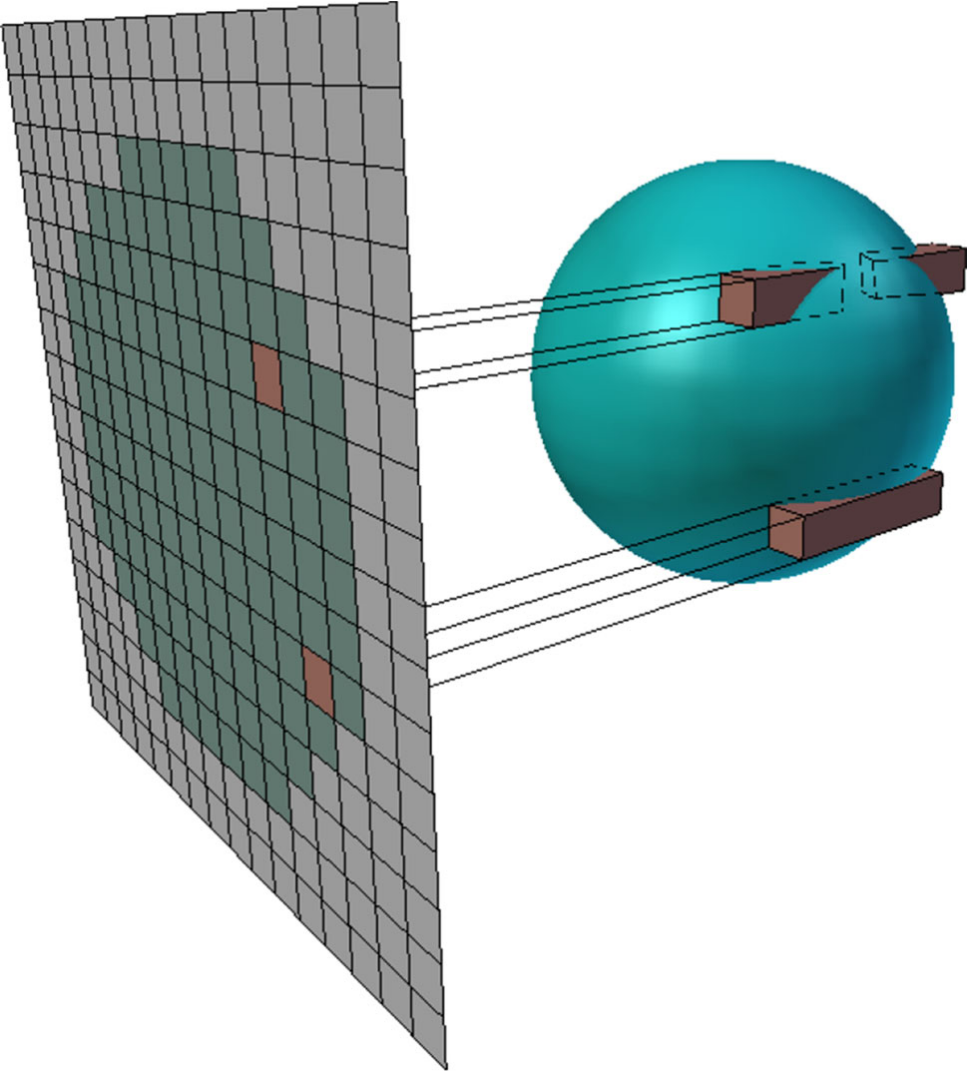
The DDS of a sphere with example cell rays from the 2D augmented view-plane grid, (top: two depth intervals, bottom: a single depth interval)

The projection plane is then discretized to a 2D grid, while the DBIs of $\hat{\Omega }$ remain continuous in the third dimension. The distance *x* between the fragment and the generating point is also discretized as the distance between their cells. We use the Manhattan distance, commonly used with grids, for faster calculations. Figure 3 illustrates the transition from the union of cylinders $\Omega '$ to discretized $\hat{\Omega }$, and Fig. 4 shows an example of the constructed discrete grid and DBIs together with the original surface the points were sampled from.

### DDS: construction and queries

The TLDI [29] construction algorithm combines the point-splatting and depth-peeling methods. The construction pipeline consists of the common three passes of point splatting: visibility, blending, and normalization  [23], augmented by three more passes to find the depth intervals contributing to the thickened layer to be extracted. The whole pipeline of full six passes is required for each depth layer, which limits the range of applications that can be performed interactively using the TLDI. It performs well with models of little depth complexity, or when only few depth layers suffice to answer most of the queries (e.g., collision detection [29]), amortizing the total runtime. However, construction time for all depth layers of complex models (e.g., indoor scenes such as large office buildings with many walls, furniture) increases quickly and thus makes it infeasible for interactive operations. Unlike the TLDI, the construction runtime of the DDS data structure proposed in this section is not dominated by the number of visibility layers, because the depth-bound intervals for all layers of the current view are constructed at once, avoiding many iterations over the input data.

**Fig. 5:**
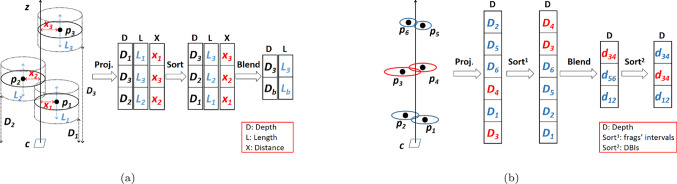
**a** The construction pipeline: A ray from grid cell *c* passes through the cylinders of three points. Each $p_i$ generates a fragment interval of length $L_i$, a distance $D_i$ from the projection plane (nearest depth), and a distance $x_i$ from the ray on that plane. The projection stage creates the fragment intervals of the points in processing order ($D_i$, $L_i$, $x_i$). The three arrays are then sorted by $D_i$. The two overlapping intervals $p_1$, $p_2$ are blended into one DBI (depth $d_b$, length $L_b$), $p_3$ become a single DBI. **b** The augmented construction pipeline: Points *p*1, *p*2, *p*5, *p*6 belong to one object, while *p*3, *p*4 belong to another. Fragment intervals are sorted by a compound key (object ID, nearest depth). Then, overlapping fragment intervals are blended. Finally, DBIs are sorted by sample depth

The main stages of the construction pipeline are (Fig. 5a illustrates this pipeline with an example):

- *C*ounting The purpose of this stage is to count the number of fragments, in total and per cell. The cylinders are projected onto the projection plane, which is similar to rendering a disk splat centered at each point in screen space, possibly covering multiple grid cells. Each fragment increments the counter of its cell in a regular frame buffer, the *c*ount buffer. The total number of fragments is then calculated from the *c*ount buffer using the *r*educe function of Thrust [20], a CUDA toolkit library for parallel computing. An *o*ffset buffer is also computed from the *c*ount buffer, using the Thrust *e*xclusive_scan function. The *o*ffset buffer holds the starting location of each fragment in the fragment interval storage buffers for each cell, and its usage is explained next.
- *P*rojection The splats are projected again to generate the fragment intervals. For each generated fragment interval, the *o*ffset buffer indicates its position in the following three storage buffers, while incrementing the *c*ount buffer:

1. A 64-bit *compound buffer* to store both the cell ID (in the 32 most significant bits) and the nearest depth (32 least significant bits)
2. A 32-bit *length buffer* to store the interval length $L=2*disk radius$
3. A 32-bit *distance buffer* to store the Manhattan distance from fragment cell to splat center cell

- *S*orting In order to keep fragment intervals consecutive per cell, we sort them by the composite key of the cell ID and the nearest depth, which are already packed in the *compound buffer*. We use the Thrust function *s*ort_by_key to sort the fragment interval indices in an *index buffer*, using the *compound buffer* as key. Using the Thrust function *g*ather, we then reorder both *length* and *distance buffers* using this *index buffer*. In our experiments, the *s*ort_by_key function performed faster than sorting cells in parallel. The reason for this is the varying number of fragment intervals per cell, which prevents effective parallelization, whereas *s*ort_by_key processes the entire array fully in parallel.
- *B*lending A traversal of the sorted fragment intervals per cell finds groups of subsequent overlapping intervals, and blends them as follows. Starting with the nearest interval $I_0$, both start and end pointers are positioned at this entry 0, and an interval *R* is initialized as $R=I_0$. At each subsequent interval $I_i$, we check whether it intersects *R*. If yes, we assign $R=R \cup I_i$, and move the end pointer to position *i*. If $I_i$ does not intersect *R*, then the fragment intervals from start to end pointer are blended into a single DBI, or in case of a single interval, copied. The traversal is then resumed with entry *i* as long as entries exist, by updating *R* as $R=I_i$, and moving both start and end pointers to *i*. This procedure creates the DBIs from fragment intervals in depth order. The depth sample of each DBI is in its center.

Out-of-core data sets have to be split into chunks for sequential processing, which requires an incremental construction of the DDS. The pipeline stages are applied to each chunk, and the resulting fragment intervals are merged with aggregated intervals in a reference structure. The DBIs are the aggregated intervals after all points are processed, when their start/end can be finally calculated as the weighted mean, for sorting them. An alternative is to sample the points using a 3D grid, scale the cylinders’ radii accordingly, and build the DDS in one go. In applications like ours, where the points are displayed on screen, the DDS uses the point subset selected by the out-of-core rendering algorithm.

**Table 1:** Comparing time and space complexity in the number of points *N* for construction, random access queries, whether neighbor elements can be retrieved with coalescing memory accesses, and space required in memory (f=load factor, L=number of layers, N=number of points, F=fragment count $\approx$ L*grid size)

Structure	Construction	R.-Access	Coal.	Space
Hashmap	**O(N)**	**O(1)**	no	O(N)f
Bintree	O(N log N)	O(log N)	no	**O(N)**
DDS	O(F log L)	**O(1)**	**yes**	**O(N)**

The time complexity of the algorithm is dominated by the sorting stage, being $O(mn.L\log {L}) \approx O(F\log {L})$, with *mn* as the discrete grid size, *L* as the number of layers, for *F* fragments. Note that, since fragments have already been binned to cells in order, the buffer is almost sorted, and just requires sorting within the layers of individual cells. As $L<< F$, the construction time of the DDS becomes near-linear. Table 1 shows that our view-dependent spatial structure is competitive with state-of-the-art structures for such purposes, as it has lower time complexity both in construction and queries than bintrees, and can exploit coalescing memory accesses, which hashmaps cannot. It also needs less or equal space than these data structures. Furthermore, it yields the connectivity of the points for free, without explicitly having to search for and connect neighbors. Time-coherent updates for changing point clouds are of the same low complexity as their proportional construction since this only touches affected grid cells.

## Occlusion-based scene exploration

**Fig. 6:**
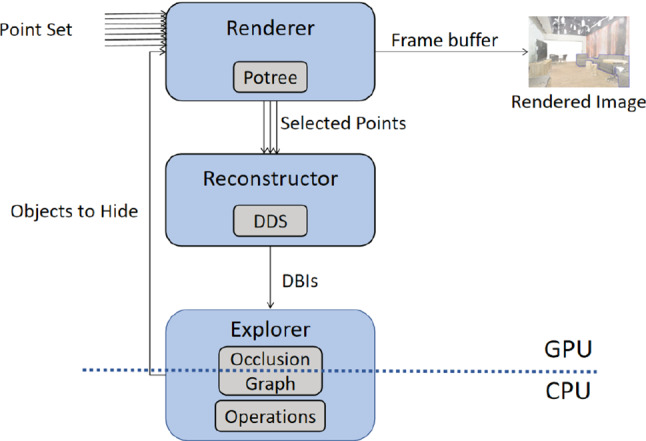
The framework components and the data flow

We demonstrate the DDS with scene exploration as application. The proposed framework consists of a rendering component, a reconstruction component, and an exploration user interface. Rendering is built upon a levels-of-detail (LOD) structure, namely Potree [34], while reconstruction and exploring are based on the DDS. The reconstructor builds the DDS, and the explorer uses the structure to construct an occlusion graph, which is queried by the exploration operations to reveal objects by hiding other objects. The list of objects marked as hidden by the exploration operations is sent to the renderer, so that their points are discarded during rendering. Both the DDS and the occlusion graph are built on the GPU, since parallelization is required for real-time performance. Post-construction, the occlusion graph is transmitted to the CPU, as searching its relatively small size is neither well parallelizable nor critical in terms of runtime. Figure 6 illustrates how the different components in the framework interact.

The input to the framework is a (potentially out-of-core) point cloud scene, which has already been scanned, labeled and stored in a format readable by Potree, namely a number of subclouds with different LODs. A prerequisite for our exploration application is the labeling of these points. Recently, several deep learning-based point cloud semantic segmentation and classification methods [22, 27, 28] have been introduced, with high accuracy rates, so that we can assume that this step can be performed efficiently in a preprocess. For our sample scene, we used a point cloud already labeled by object categories. Points of the same category are further clustered into individual objects based on proximity, as explained in more detail in Sect. 5.

### Reconstruction

The rendering component selects a number of visible subclouds (octree nodes) to load into the GPU at render time, based on the current view. In Potree, points are displayed as soon as the first, coarser, nodes are loaded, while finer subclouds are loaded in subsequent frames to refine the rendered image. When moving the camera, this process is repeated. Thus, the subset of points rendered in the current frame often changes a lot from the previous frame. Each point is rendered as a splat, with its radius proportional to the level of detail (LOD) of its subcloud. The reconstruction component builds a DDS of the scene upon user request, using the available selected points at request time. We assign the splat radius of the points as their cylinder radius, in order to achieve a mostly hole-free reconstruction. In order to create the occlusion graph from the user’s view, we build the DDS according to these camera parameters, with the view plane as its projection plane, and the same rendering perspective projection. The 2D grid has the same resolution of the render frame buffer as default. Reducing the grid resolution is easily possible, trading off little precision for faster construction.

#### Per-object reconstruction

For occlusion detection, we require the depth samples of the DDS to be labeled with object IDs so we can determine the relations between entire objects. This requires some augmentation of the DDS construction process.

First, we have to avoid blending the fragment intervals of different objects. Thus, fragment intervals have to be sorted by cell ID, object ID and depth. CUDA supports variables of up to 64 length; therefore sorting can only be performed in a single pass if the three attributes fit in a 64 bits composite key. This is possible only up to the case of a full HD resolution and $2^{11}$ objects (or tradeoffs between those). Otherwise, we sort the fragment intervals two times, first by depth, and then by a composite key of cell ID and object ID using Thrust *stable_sort*, which maintains the relative order of entries with similar key values. The results of the blending stage are then consecutive DBIs per object for each cell. After the DBIs have been generated on a per-object basis, they are still sorted by object ID before depth for each cell. Therefore, we perform an additional sort with the original composite key (cell ID, depth) in order to correct the depth ordering. Figure 5b illustrates the augmented pipeline with an example.

### Occlusion detection with the DDS

We can now determine the occlusion relations between objects by looking at the order of the sorted depth samples per cell. Note that per cell we only have to consider the front-most (nearest) depth sample for each object. Therefore, for each label we only consider the nearest (i.e., first encountered) depth sample and ignore those that follow. If we encounter a nearest depth sample labeled X and then a nearest depth sample labeled Y, we mark object X as occluding Y (plus all other objects for which a nearest depth sample follows).

**Fig. 7:**
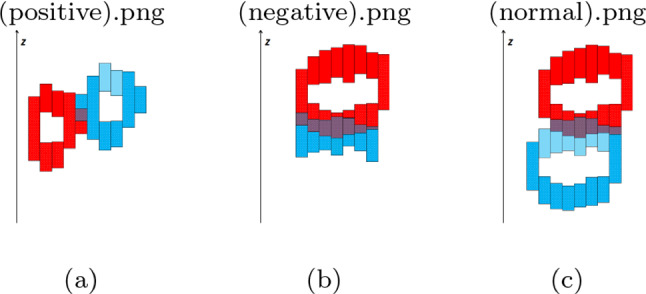
Cross sections of the DDS from top view, for different cases of occlusion. The nearest DBIs are dotted. **a** Nearest DBIs in the occluder and occludee intersect at the boundaries extensions of the two objects. Due to the intersection, no occlusion is reported, otherwise it would be a false positive case. **b** A false negative case: The occluder is a thin object with only one layer, all its DBIs intersect with the nearest DBIs of the occludee. **c** A regular case, with two layers in both occluder and occludee. Occlusion is detected, because there is at least one nearest DBI in the occluder that precedes a nearest DBI in the occludee, so they do not intersect

A common case of obvious false positives can happen at the boundaries, where the nearest depth samples of two adjacent but non-occluding objects are projected into the same cell, such as in Fig. 7a. Since the DBIs of such depth samples are usually close in depth and intersect in their range, we manage to exclude such false positives by ignoring depth samples in that case. This condition could result in false negatives when the occluder and occludee are close along the depth direction and the occluder consists of only one layer, so that the nearest DBIs of the two objects intersect in all cells, such as in Fig. 7b. But this is a very rare case. In real scenes, most of the objects have more than a single depth layer (even, e.g., a thin wall), and the nearest DBIs of the occluder and occludee are separated by DBIs of other layers of the occluder, as in Fig. 7c.

In point clouds scanned from real-world scenes, data is often missing or sparse at regions that are invisible or hardly visible from scan positions. Our occlusion finding algorithm is robust to such data deficiencies, because only data that is actually present forms layers.

### Occlusion graph and visibility layers

From these occlusion relations we can now construct the occlusion graph with objects as nodes and occluder–occludee relations as directed edges. The cells’ DBIs are traversed in parallel, each cell with a thread, to accumulate all occlusions tuples (occluder, occludee) in an array. The tuples are sorted by Thrust *sort*, duplicates are removed by Thrust *unique*, and the graph is constructed as a list of objects with each object keeping a pointer to a set of its occluders.

**Fig. 8:**
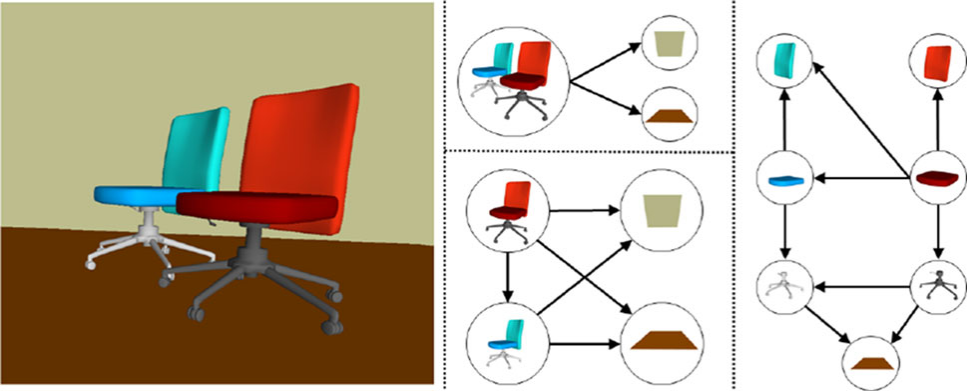
The complexity of the occlusion graph of a scene depends on the segmentation granularity. *L*eft Scene of two chairs, floor and wall. Occlusions graphs: *C*enter top For nearby objects of the class grouped as single object. *C*enter bottom For object-level segmentation. *R*ight: For part-level segmentation. Here, all the chairs parts also occlude the wall (omitted for simplicity)

The number of non-overlapping objects (depending on the level of segmentation) determines the complexity of the occlusion graph; the more objects, the more complex is the graph. In indoor environments, a segmentation can break down the scene into individual objects, further into semantically defined parts, or even into a convex decomposition [1] of parts. Coarser levels are also possible, such as segmenting the scene into rooms. Figure 8 shows a simple example scene and corresponding occlusion graphs of different segmentation levels. In case more than one level of segmentation is provided, e.g., encoded in per-level bitsets of the object ID, the user can control the graph complexity and move from one level to another interactively. In our experiments, we segment the scene into objects, but nearby objects of the same class may be grouped together if they overlap, as will be explained in more detail in Sect. 5.

In order to reveal an object, we just have to remove its occluders. Occluders can also be arranged in visibility layers, so that the user can browse through them, instead of hiding all occluders at once. We define the visibility layers as an ordered list $(l_0,l_1,...)$ of unordered sets, such that $l_0$ is the set of entirely unoccluded objects, and $l_i$ is the set of objects not occluded by any objects except those in $l_{0..i-1}$, and so on. The quadratic-time layering algorithm searches for totally non-occluded nodes and adds them to $l_0$, and repeats this for the next layers.

**Fig. 9:**
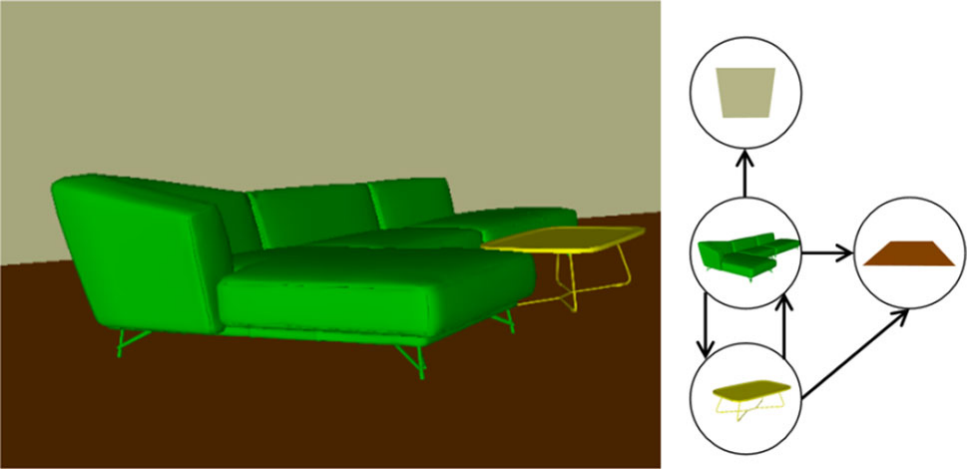
A cycle is encountered while assigning objects (all occluded) to a layer. The least occluded object (table) is added to the layer

A sequence of objects may form a cycle in the occlusion graph, where each object occludes the next in a loop. Separating this set of objects into several visibility layers is impossible, as each of them will then be always occluded by another one in that set. If the graph contains at least one such cycle, even if it is as small as two nodes, then the layering algorithm will fail to find any non-occluded nodes to add to $l_i$ at some *i*. When this happens, we determine the object least occluded (that is, the object with the fewest occluded nearest depth samples) and add it to $l_i$. In case the selected object is not part of the cycle, the cycle persists, and is encountered again in the next layer(s), where the process is repeated. Figure 9 shows an example of an occlusion cycle encountered during the layering process. Selecting the least occluded objects is a heuristic, based on the fact that our system explores objects by revealing them. Other heuristics are possible, such as selecting the nearest object to the camera, or the one occluded by the least number of objects. The user can select the preferred strategy.

### Exploration tools

Once the DDS has been constructed for the current view, the user can explore novel exploration operations until they move the camera. Desired objects are highlighted by rendering their boundaries with a distinct shade, e.g., blue. The list of boundary pixels is sent to the rendering component to update the current frame. Being able to expose (partially, or even multiply) occluded objects can help the users to faster understand environments, especially in cluttered indoor scenes. We propose the following functions:

- *Click to Reveal*: The user selects a partially visible object and clicks to hide all its occluders.
- *Reveal by Class*: In case objects are categorized (e.g., chairs, tables, etc.), the user chooses to reveal all objects of a specific class.
- *Hide with Occluders*: Selecting an object and hiding it is a simple operation that does not need occlusion information. This advanced operation hides the selected object together with all its occluders. This operation is a composite of ’Click to Reveal’ an object, and then applying ’Hide’ to it. It can be useful when the user wants to hide a whole room, for example, so that they can select the wall in order to hide it plus its occluders.
- *See through*: The objects behind the visible object along the cursor position are displayed on a panel on the right, sorted by depth. In dense environments, the number of displayed occludees can be controlled by the user. This operation is useful when the user wants to explore the scene without hiding objects. It also reveals fully occluded objects.
- *Browse Occluders*: Some interesting occluders of an object could be fully occluded themselves, and if the user would apply the *Click to Reveal* operation on that object, those would be hidden. Instead of hiding all occluders of an object at once, this operation arranges the occluders in their visibility layers, and the browsing operation is then applied to objects per visibility layer. This operation is most useful when the user is searching for a specific item.
- *Browse Layers*: We arrange all objects in the scene (instead of just occluders of a single selected object as above) in visibility layers, and can then browse forward and backward through them. This function is useful for quick understanding of small scenes.

**Fig. 10:**
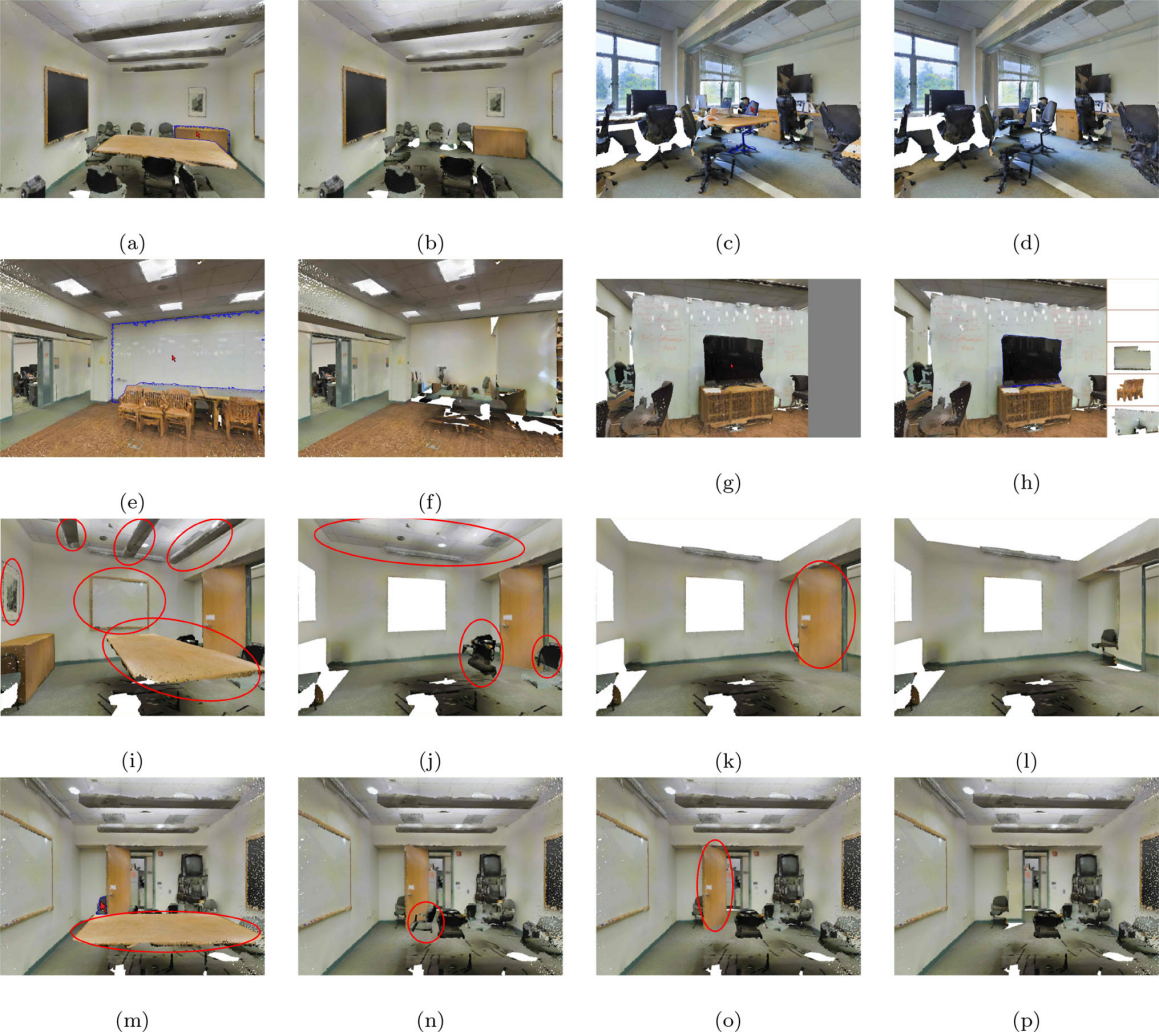
Exploration functions, always before/after click. **a**, **b** *Click to Reveal* reveals the selected object. **c**, **d** *Reveal by Class* reveals the selected object together with all other objects of the same category. **e**, **f** *Hide with Occluders* hides the selected object together with its occluders. **g**, **h** *See through* displays the occluded objects along the cursor. **i**–**l** *Browse Layers* browses the layers of the scene. In each subfigure of the browsing functions, the objects to be removed the next subfigure are enclosed in red. **m**–**p** *Browse Occluders* browses the layers of objects occluding the selected object. Objects to be removed are also enclosed in red

We keep an array of hidden objects that is updated after each operation and sent to the rendering component, so that it can discard the points of these objects during rendering. Alternatively, such objects could be turned semi-transparent. Figure 10 shows examples of the *Click to Reveal*, *Reveal by Class*, *Hide with Occluders*, *See through*, *Browse Occluders* and *Browse Layers* operations applied to the objects of a scene.

**Fig. 11:**
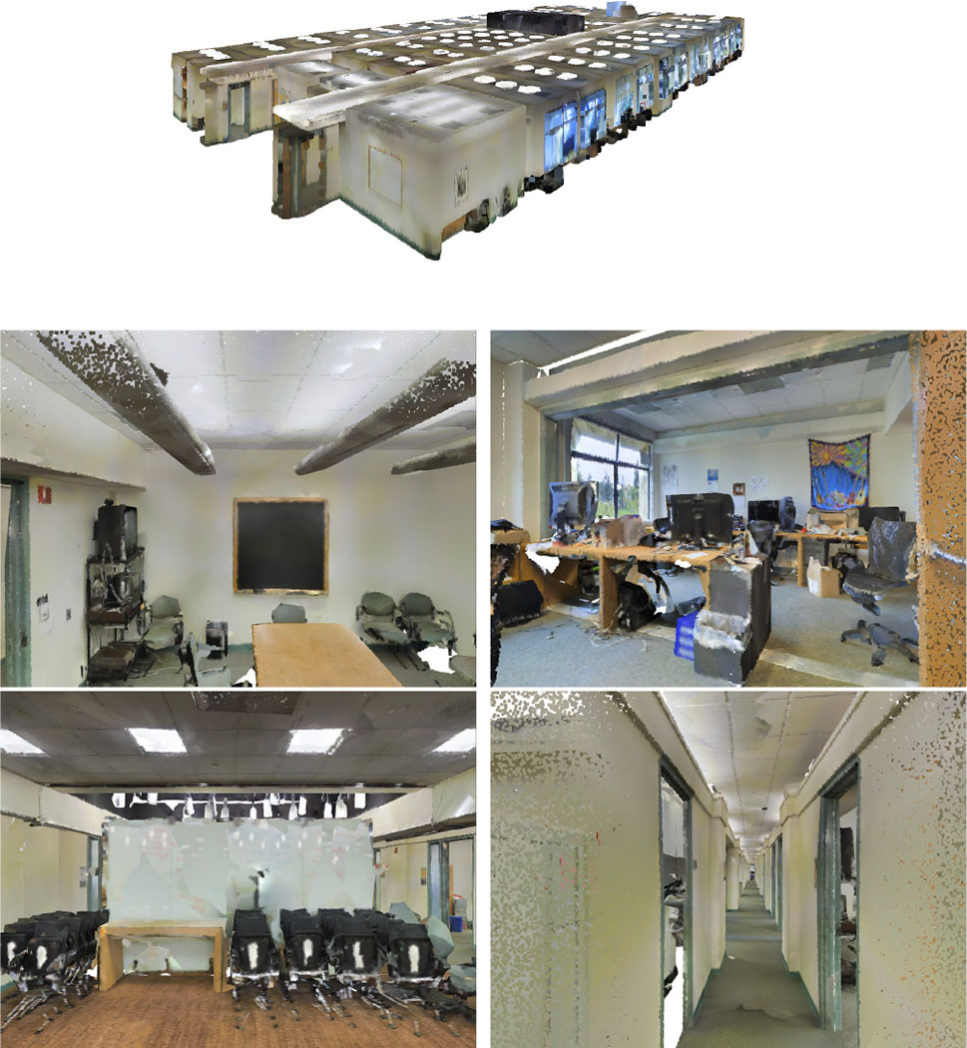
The model and the four views used in the quantitative comparison. *T*op Top view of the model. *B*ottom View 1–4 in reading order

**Table 2:** DDS vs TLDI construction time with respective graphs

view id	#points	DDS $(512^2)$	DDS $(256^2)$	TLDI	DDS $(512^2)$ Graph	DDS $(256^2)$ Graph	TLDI Graph
1.1	522k	115	41	4623	26.8	10.5	7.07 minutes
1.2	773k	125	45	4872	26.5	10.1	7.08 minutes
1.3	1.7M	151	58	3607	27.7	11.1	7.35 minutes
1.4	2.3M	149	59	3649	29.4	11.6	7.61 minutes
2	1.9M	126	49	3565	20.1	8.8	7.44 minutes
3	3.6M	168	72	4013	21.4	9.5	7.05 minutes
4	5.4M	205	97	3529	38.1	14.9	7.12 minutes

Timings in milliseconds, except for TLDI Graph

## Evaluation

We used the open source software Potree [34] as the rendering component. The DDS and exploration tools were implemented using C++ and CUDA, as well as OpenGL shaders to show/hide objects. All experiments were performed on an AMD Ryzen 7 3700X 8-Core CPU, with 64 GB RAM, and GeForce RTX 2070 SUPER GPU. The framework was tested on a large indoor scene (http://buildingparser.stanford.edu/) with more than 30 rooms and 40M points. The points of the scene are labeled as common indoor categories, such as chairs, tables, etc., however they are not grouped together as objects. For the purpose of our experiment, we performed a quick heuristic segmentation of points in each category in a preprocess: First, a seed point is randomly chosen, then points of the same category in its 10-nearest-neighbors set are added recursively as long as points can be added. This is repeated for unvisited points until all points are grouped in sets. A set can contain the points of an object or a part of an object (e.g., chair arm). Next, sets of less than 500 points are marked as small, and joined with the nearest non-small set of the same category. Some close objects of the same category (e.g., chairs) are joined in a single set, which we found acceptable in our experiments.

*DDS vs TLDI:* We compared the computation times of the DDS (grid sizes 256$\times$256 and 512$\times$512) against the TLDI (grid size 512$\times$512). Four different views (see Fig. 11), one of them with different resolution, were taken from the model, on which both the DDS and TLDI were applied. Table 2 shows the construction time of the two structures in milliseconds. For the particular application of finding occlusions, it is sufficient to compute the first layer only of the TLDI. Nevertheless, the $512^2$ DDS construction time is about 20 to 40 times faster than the TLDI. This is due to the fact that the TLDI is built per object, and the scenes we processed consist of about thousand objects (see Table 3). Table 2 also shows that finding occlusions with TLDI is several magnitudes slower than finding them using the occlusion graph with the DDS, because the TLDI layers of the objects have to be compared in pairs, which results in quadratic runtime, making detecting occlusions with the TLDI infeasible for any but very small scenes.

**Table 3:** Occlusion graph sizes for DDS with $512^2$ and $256^2$ grid resolution (N=nodes, E=edges)

view	#N $(512^2)$	#N $(256^2)$	#E $(512^2)$	#E $(256^2)$
1.4	1512	1513	55072	53865
2	865	866	28238	27840
3	1029	1029	26233	26327
4	1658	1659	64479	63417

**Table 4:** Runtime of the DDS $(512^2)$ construction stages (in milliseconds, and percent, averaged over views 1–4)

Count	Proj	Sort Frag	Blend	Sort DBIs	Total
*O*(*F*)	*O*(*F*)	$O(F \log L)$	*O*(*F*)	$O(F \log L)$	
11	32	60	11	6	120
9.2%	26.6%	50.0%	9.2%	5%	100%

*DDS runtime analysis:* Table 2 also shows that the construction time of a downsized $(256^2)$ DDS and the associated occlusion graph are both reduced to about 35$\%$–40$\%$, compared to the $(512^2)$ DDS. Construction times for each resolution are very close, independently of the number of points, which supports our claim that DDS construction time is mostly output sensitive.

Table 4 details the complexity and runtime of each stage in the augmented DDS $(512^2)$ construction pipeline. Sorting fragments is the dominant stage, and projecting disks is next, with less than half the runtime. Sorting the DBIs takes relatively short time, because the number of DBIs is usually 5–10 times smaller than the number of fragments. Other than the sorting of the almost-sorted buffer, the linear stages sum up to almost 50% of the total runtime.

Reduced resolution comes with reduced precision. However, Table 3 shows that the error in occlusions detected (represented by differences of edge count in the occlusion graph) is $2\%$ or less.

## Conclusion and potential applications

In this paper, we presented the—TLDI-based—DDS, a view-dependent data structure with output sensitive runtime and near-linear time complexity for practical cases that approximates a tight surface bound from point sets in real time. Since the separate layers of the surface w.r.t. visibility from the current view are already sorted in this structure, occlusions are easily detected with little computational effort. This is both useful and efficient for users to locate hidden objects in cluttered scenes.

The DDS as time- and space-efficient structure has potential for many additional purposes, and we intend to continue studying it in different applications. The following are potential uses of the structure:

- *Point cloud data structure:* Instead of resampling the depth values of the original points into DBIs, the original samples could be kept in a list linked to their DBIs. This enables speeding up operations on the points’ neighborhoods, such as kNN search.
- *Voxel-based representation:* The DDS is quite similar to voxel structures, except that its bounding intervals along one axis, e.g., depth, are continuous and do not have to be aligned. This offers both more flexibility and resolution. Like grids, the DDS can function as an intermediate representation of a model, and then be meshed, also easily more coarsely.
- *Bounding volume:* The structure also serves as a tight bounding volume, which is useful for, e.g., simulations and shape deformation, among others.
- *Compact representation:* As the bounding intervals have no fixed depth, the DDS encodes spaces much more efficiently than a grid or voxels. This can be exploited in change detection, where storing the occupied space between surfaces would require too much space with traditional hierarchical data structures.
- *Visibility-based geometry filter:* The DDS can prune the nodes selected by a LOD structure for rendering by discarding nodes occluded by the visible layers.

## Supplementary Information

Below is the link to the electronic supplementary material.

Supplementary material 1 (mp4 10547 KB)

Supplementary material 2 (mp4 2781 KB)
